# Prevalence of mental disorders among middle school students in Shaoxing, China

**DOI:** 10.1186/s12991-023-00463-0

**Published:** 2023-08-28

**Authors:** Shuangyi Pei, Xiaoting Wu, Weijiang Ye, Linqi Fang, Haoqiang Zhang, Fanghua Zhou, Xiaofei Du, Xinyi Cao, Shengnan Ma, Yuanchu Li, Shuwen Xi, Pingping Xu

**Affiliations:** 1 Zhejiang Rehabilitation Medical Center (Rehabilitation Campus of the Third Affiliated Hospital of Zhejiang Chinese Medical University), Hangzhou, China; 2 Tangshan Vocational and Technical College, Tangshan, China

**Keywords:** Mental disorder, Prevalence, Gender, Epidemiology, Obsessive–compulsive disorder, Major depressive disorder

## Abstract

**Background:**

In China, adolescents account for about a quarter of those treated for mental disorders each year, and adolescent mental health issues have become a social hotspot. Although several epidemiological surveys of mental disorders have been conducted in China, no study has yet focused on the prevalence of mental disorders among adolescents in a certain region of Zhejiang.

**Methods:**

In the first stage, 8219 middle school students aged 12–18 years in a city of Zhejiang Province (Shaoxing) were screened with the mental health screening checklist. In the second stage, participants who screened positive were tested with the Mini International Neuropsychiatric Interview for Children and Adolescents (MINI-KID) and the Diagnostic and Statistical Manual of Mental Disorders, Fourth Edition (DSM-IV). Then, the prevalence of mental disorders were calculated.

**Results:**

The overall prevalence in this population was 12.4%, with prevalence rates exceeding 20% in both the 17- and 18-year-old age groups. The most common mental disorders were obsessive–compulsive disorder (OCD) (9.1%) and major depressive disorder (MDD) (8.9%).

**Conclusions:**

Mental disorders are common among middle school students, and girls are at higher risk than boys. As the most prevalent mental disorders, OCD and MDD should receive timely attention, especially for upper grade students.

## Background

Mental disorders refer to a diverse range of conditions that can impact an individual's thinking, feeling, behavior, and overall well-being, which can arise from a confluence of genetic, environmental and psychological factors [1]. Some of the most common mental disorders include major depressive disorder (MDD), anxiety disorders (AD), and obsessive–compulsive disorder (OCD).

Globally, epidemiological studies estimate the prevalence of any mental disorder to be between 3.5% and 38.3% [1, 2]. Not only do mental disorders have a serious impact on the quality of life of those suffering from them, but they also carry impose a substantial socio-economic burden. It is noteworthy that adolescents with mental disorders are more likely to develop psychological disorders in adulthood, as well as have more difficulty in dealing with financial and social problems, and experiencing greater challenges in managing family relationships and friendships [3]. As a result, researches focusing on adolescent mental disorders have increased in recent decades, and the issue of adolescent mental health has emerged as a global challenge. According to data from 41 studies from 27 countries in 2015, it was determined that at least 11–16% of children and adolescents suffer from one or more mental disorders [2]. However, despite the high prevalence rates, many studies have shown that the number of adolescents currently receiving treatment for mental disorders is much lower than the number of universal screening [4–6]. These suggest that a considerable number of adolescents are not receiving adequate professional diagnosis and treatment for their mental health conditions. Hence, it is critical to increase research and awareness of adolescent mental disorders to address the above problems and improve the quality of life for affected individuals. Furthermore, it is essential to provide accessible and effective mental health services and supports to guarantee that adolescents' mental health problems are identified and managed promptly and adequately.

In China, mental and psychiatric disorders rank first in the country's total burden of disease, with adolescents accounting for about a quarter of those treated for mental disorders each year [7, 8]. The Institute of Psychology of the Chinese Academy of Sciences conducted a survey in 2020 to assess the levels of various dimensions of mental health among China's youth population. The survey results indicate that the attainment rate of mental health literacy among the China's youth is notably low, measuring at 14.24% [7, 8]. In addition, the China Youth Development Report, published in December 2019, estimates that a minimum of 30 million children and young people under 17 years of age in China are experiencing diverse emotional and behavioural difficulties [7, 8].

What is more, the imbalance between the psychological development and physical development of adolescents makes them more prone to difficulties in psychological integration, leading to negative states of mind, such as worry, anxiety, depression, loneliness and low self-esteem, and thus mental health problems. In recent years, the number of children and adolescents experiencing mental problems worldwide has been on the rise and is of increasing concern to families, schools and society. Although there are more research studies on the mental health status of adolescents, the findings are not consistent across different geographical areas and different levels of economic development [7, 8].

The aim of this study was to investigate the prevalence and distribution of common mental disorders in Shaoxing, China. A two-stage epidemiological survey was conducted. The mental health status of a total of 8219 students in eight middle schools was screened using a questionnaire, and then the Diagnostic and Statistical Manual of Mental Disorders, Fourth Edition (DSM-IV) criteria were used to determine the diagnosis to clarify the current status of mental disorders among these students. This study will contribute to the future provision of medical resources, health policies and prevention strategies.

## Methods

### Participants

Shaoxing, a city located in Zhejiang Province, was chosen for this study. Eight secondary schools were randomly selected and a total of 8219 participants were included. Of these, 205 refused to participate, 291 did not complete the questionnaire, 75 of those who completed the questionnaire had missing data (more than 20% of the questionnaire was incomplete), and 131 were out of age. Ultimately, a total of 7493 eligible questionnaires (91.2%) were obtained for this study.

### The screening tool

The Mental Health Screening Checklist (MHSC, self-rated version) completed by the students was used, which consists of five scales: symptom inventory 90 (SCL-90), Self-rating depression scale (SDS), Self-rating anxiety scale (SAS), Yale-Brown obsessive compulsive scale (Y-BOCS) and Pittsburgh sleep quality index (PSQI). The SCL-90 is a self-reported mental health instrument proposed by Derogatis and is widely used to detect clinical psychiatric symptoms and mental health conditions [9–11]. It can distinguish healthy individuals from those with psychosis and has good reliability and validity for assessing the mental health of individuals as well as the overall assessment of mental health in different groups. Both the SDS and SAS cover 20 items specific to psychological and physical conditions and are used as self-measures of depression and anxiety, respectively, rated by the respondent on the basis of the past week, with higher scores indicating poorer psychological conditions [12, 13]. The Y-BOCS is considered to be the gold standard for assessing the severity of OCD and consists of a symptom checklist and 10 items [14]. PSQI is one of the most widely used measures of sleep quality, consisting of seven sleep parameters to assess different sleep problems, and is valuable in predicting the risk of depressive symptoms [15].

### Diagnostic criteria and tools

Two types of interviews were conducted sequentially based on the Mini International Neuropsychiatric Interview for Children and Adolescents (MINI-KID) and DSM-IV criteria [16, 17]. The MINI-KID was used for a brief formal psychiatric examination and its scores were provided to the psychiatrist conducting the DSM-IV interview as a reference for the final diagnosis.

### Assessment procedure

**Fig. 1:**
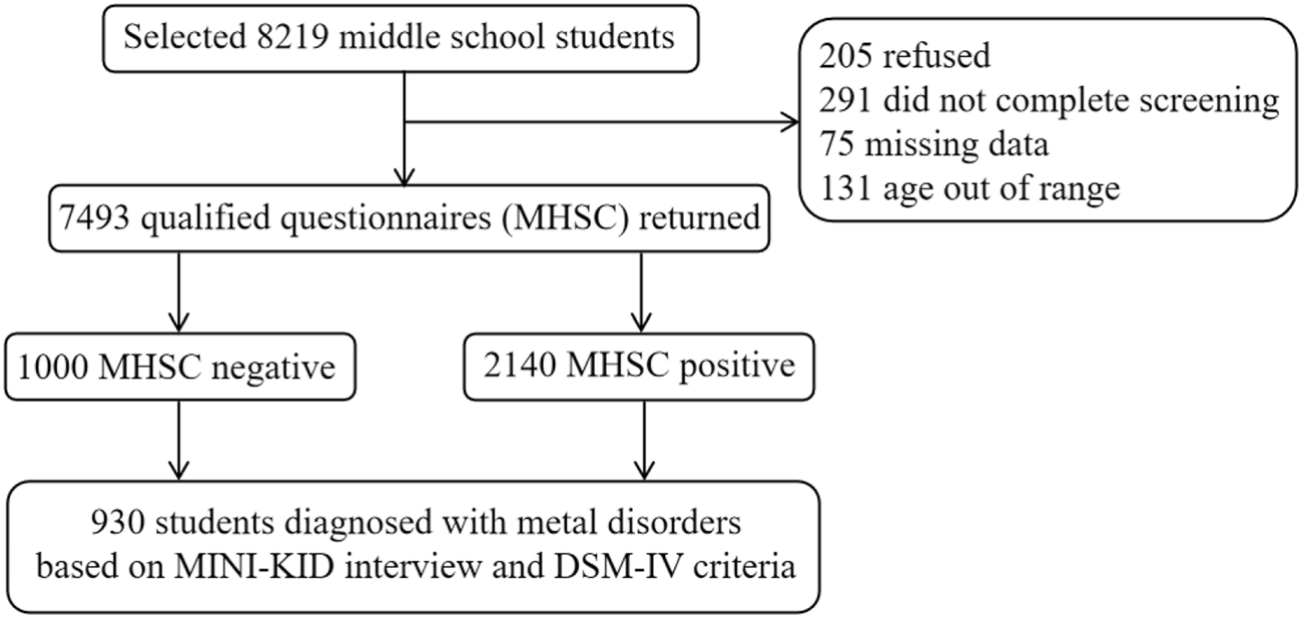
Study flow chart

The project was approved by the Ethics Review Committee of the Zhejiang Rehabilitation Medical Center. This study consisted of two steps, with the first step using MHSC as the investigative tool. The survey was conducted by a team of psychiatrists, qualified doctors and medical students. The survey data were initially processed by a statistician and after excluding items with incomplete or confusing responses, all individuals with complete MHSC records were analyzed. Of the 7723 participants who completed the questionnaire, 7493 were eligible, with 28.6% of the students scoring positive, i.e., scoring positive on at least one of the five scales included in the MHSC. Then, the second step included all participants with MHSC positive, as well as 1000 randomly selected participants with a negative result, who were chosen to assess the false-negative rate of MHSC. The second step consisted of two interviews. The psychiatrist first conducted a semi-structured interview based on the MINI-KID, followed by a DSM-IV interview to confirm the diagnosis, and finally a total of participants were identified as having at least one psychiatric disorder. The procedure is illustrated in Fig. 1.

### Statistical analysis

Data analysis was performed using SPSS 25.0 software. Prevalence, frequencies and 95% confidence intervals (CIs) were estimated for mental disorders and comorbidities. The gender, age and regional distribution of the sample, as well as the prevalence of different mental disorders in different age groups, were assessed and subsamples were compared using *T* tests and Chi-square tests. All statistical tests were two-tailed with a significance level of 0.05. Logistic regression analysis was used to study the age group, gender, region, prevalence and the interaction among them.

## Results

A total of 8,219 students participated in the MHSC questionnaire and 7493 qualified questionnaires eventually returned, with a gender distribution of 3702 males and 3791 females. Among them, 2140 students were found to have psychological problems, with a detection rate of 28.6% (95% CI 27.5–29.6). The prevalence rate of girls (31.9%, 95% CI 30.4–33.4) was significantly higher than that of boys (25.1%, 95% CI 23.8–26.5) (*χ*^2^ = 41.736, *p* < 0.01; Table 1). Furthermore, as shown in Table 1, the prevalence rate was 23.5% (95% CI 21.9–25.0) for 12–13 years, 29.8% (95% CI 28.2–31.4) for 14–15 years and 36.9% (95% CI 34.2–39.5) for 16–18 years, and there was a statistically significant difference in the detection rate of metal disorders by age group (*χ*^2^ = 82.768, *p* < 0.01).

**Table 1:** Prevalence, gender and age of mental disorders among 12–18-year-old students based on MHSC

Variable	Sample	Rate (%)	95% CI (%)	*χ*^2^	*p* value	Cramer’s *V*
Prevalence	7493	28.6	27.5–29.6			
*Gender*						
Boys	3702	25.1	23.8–26.5	41.736	0.001**	0.075
Girls	3791	31.9	30.4–33.4			
*Age*						
12–13	2898	23.5	21.9–25.0	82.768	0.001**	0.105
14–15	3318	29.8	28.2–31.4			
16–18	1277	36.9	34.2–39.5			

*p < 0.05; **p < 0.01

Of the 7493 students, 2140 participants with MHSC positive and 1000 participants with MHSC negative were included in the secondary interview. A total of fourteen types of mental disorder were diagnosed in 930 children through the MINI-KID and DSM-IV interview. Among these mental disorders, obsessive compulsive disorder (OCD) had the highest prevalence (9.1%, 95% CI 8.4–9.7), followed by major depressive disorder (MDD; 8.9%, 95% CI 8.3–9.6), anxiety disorders (AD; 4.8%, 95% CI 4.3–5.3), attention–deficit hyperactivity disorder (ADHD; 2.5%, 95% CI 2.1–2.8), oppositional defiant disorder (ODD; 1.9%, 95% CI 1.6–2.2) and sleep disorder (SD; 1.9%, 95% CI 1.6–2.2) (Table 2).

**Table 2:** Point prevalence of mental disorders in 12–18-year-old students (*n* = 7493)

DSM-IV Disorders	*N*	%	95% CI (%)
Any disorders	930	12.4	11.7–13.2
Major depressive disorder	670	8.9	8.3–9.6
Anxiety disorder	360	4.8	4.3–5.3
Obsessive compulsive disorder	680	9.1	8.4–9.7
Sleep disorder	140	1.9	1.6–2.2
Attention–deficit hyperactivity disorder	187	2.5	2.1–2.8
Oppositional defiant disorder	142	1.9	1.6–2.2
Tic disorder	67	0.9	0.7–1.1
Substance use disorder	60	0.8	0.6–1.0
Mania or hypomania	37	0.5	0.3–0.7
Social phobia	97	1.3	1.0–1.6
Post-traumatic stress disorder	15	0.2	0.1–0.3
Bulimia nervosa	60	0.8	0.6–1.0
Anorexia nervosa	15	0.2	0.1–0.3
Psychotic disorder	6	< 0.1	0–0.1

^*^p < 0.05; **p < 0.01

As shown in Table 3, the overall point prevalence rate for boys was 10.8% (95% CI 9.8–11.7) lower than the 14.0% (95% CI 12.9–15.1) for girls. Compare to boys, the prevalence of the MDD, AD, OCD, SD, bulimia nervosa (BN) and anorexia nervosa (AN) was significantly higher in girls, at 10.0% (OR 0.763), 5.5% (OR 0.720), 10.0% (OR 0.797), 2.3% (OR 0.599), 1.4% (OR 0.112) and 0.3% (OR 0.157), respectively. However, prevalence rates for ADHD, tic disorder (TD), substance use disorder (SUD) and mania or hypomania (MA) were higher for boys, at 3.5% (OR 2.447), 1.5% (OR 4.749), 1.5% (OR 9.338) and 0.8% (OR 4.416), respectively. There was no gender difference in the risk of ODD, social phobia (SOP), post-traumatic stress disorder (PTSD) and psychotic disorder (PD) (*p* > 0.05).

**Table 3:** Comparison of the prevalence of mental disorder groups between males and females

DSM-IV disorders	Male (*n* = 3702)	Female (*n* = 3791)	Odds ratio (95% CI)	*p* value
Any disorders	10.8 (9.8–11.7)	14.0 (12.9–15.1)	0.738 (0.642–0.848)	0.001**
MDD	7.8 (7.0–8.7)	10.0 (9.1–11.0)	0.763 (0.650–0.865)	0.001**
AD	4.1 (3.4–4.7)	5.5 (4.8–6.3)	0.720 (0.581–0.892)	0.003**
OCD	8.1 (7.2–9.0)	10.0 (9.0–11.0)	0.797 (0.680–0.934)	0.005**
SD	1.4 (1.0–1.8)	2.3 (1.8–2.8)	0.599 (0.424–0.827)	0.003**
ADHD	3.5 (2.9–4.1)	1.5 (1.1–1.9)	2.447 (1.783–3.357)	0.000**
ODD	2.2 (1.7–2.6)	1.6 (1.2–2.0)	1.328 (0.951–1.857)	0.095
TD	1.5 (1.1–1.9)	0.3 (0.1–0.5)	4.749 (2.539–8.883)	0.000**
SUD	1.5 (1.1–1.8)	0.2 (0.0–0.3)	9.338 (4.013–21.731)	0.000**
MA	0.8 (0.5–1.1)	0.2 (0.0–0.3)	4.416 (1.937–10.067)	0.000**
SOP	1.2 (0.9–1.6)	1.3 (1.0–1.7)	0.923 (0.618–1.378)	0.694
PTSD	0.1 (0.0–0.3)	0.3 (0.1–0.4)	0.511 (0.175–1.497)	0.213
BN	0.2 (0.0–0.3)	1.4 (1.0–1.8)	0.112 (0.048–0.261)	0.000**
AN	0.1 (0.0–0.1)	0.3 (0.2–0.5)	0.157 (0.035–0.697)	0.005**
PD	0.1 (0.0–0.2)	0.1 (0.0–0.1)	2.049(0.375–11.195)	0.398

^*^p < 0.05; **p < 0.01

MDD, major depressive disorder; AD, anxiety disorder; OCD, obsessive–compulsive disorder; SD, sleep disorder; ADHD, attention–deficit hyperactivity disorder; ODD, oppositional defiant disorder; TD, tic disorder; SUD, substance use disorder; MA, mania or hypomania; SOP, social phobia; PTSD, post-traumatic stress disorder; BN, bulimia nervosa; AN, anorexia nervosa; PD, psychotic disorders

It is noteworthy that the prevalence of overall mental disorders increases with age, as shown in Table 4. The prevalence of mental disorders was upwards of 20% in the 17–18-year-old age group relative to other age groups (21.9%, 95% CI 17.7–26.2 and 21.8%, 95% CI 13–30.7, respectively), while the lowest prevalence in the 12-year-old age group at less than 10% (8.7%, 95% CI 6.9–10.4). The prevalence of MDD and anxiety disorders was highest among participants aged 18 years (18.4%, 95% CI 10.1–26.7 and 10.3%, 95% CI 3.8–16.9, respectively), and the prevalence of OCD, SD and SUD was highest among participants aged 17 years (20.5%, 95% CI 16.4–24.7, 3.0%, 95% CI 1.3–4.8 and 3.0%, 95% CI 1.3–4.8, respectively). Inconsistently, the younger the age, the higher the risk of ADHD, ODD and TD, with prevalence rates of 5.2% (95% CI 3.8–6.6), 3.5% (95% CI 2.3–4.6) and 3.3% (95% CI 2.1–4.4), respectively, in the 12-year-old group (Table 4).

**Table 4:** Age distribution of several common mental disorders

Age	12 years	13 years	14 years	15 years	16 years	17 years	18 years	Sum
Any disorders	8.7 (6.9–10.4)	10.9 (9.5–12.2)	12.1 (10.7–13.5)	12.8 (10.9–14.7)	15.5 (13.0–18.0)	21.9 (17.7–26.2)	21.8 (13.0–30.7)	12.4 (11.7–13.2)
MDD	7.3 (5.7–9.0)	7.9 (6.7–9.1)	8.2 (7.0–9.4)	8.3 (6.7–9.8)	11.6 (9.4–13.8)	16.7 (12.9–20.6)	18.4 (10.1–26.7)	8.9 (8.3–9.6)
AD	3.5 (2.3–4.6)	4.2 (3.3–5.1)	5.1 (4.2–6.0)	4.4 (3.2–5.5)	5.7 (4.1–7.3)	7.9 (5.2–10.7)	10.3 (3.8–16.9)	4.8 (4.3–5.3)
OCD	5.8 (4.3–7.3)	7.3 (6.1–8.5)	8.0 (6.9–9.2)	8.9 (7.3–10.5)	13.8 (11.5–16.2)	20.5 (16.4–24.7)	19.5 (11.0–28.0)	9.1 (8.4–9.7)
SD	1.1 (0.5–1.8)	1.5 (0.9–2.0)	2.3 (1.7–3.0)	1.6 (0.9–2.4)	2.3 (1.3–3.3)	3.0 (1.3–4.8)	2.3 (0.0–5.5)	1.9 (1.6–2.2)
ADHD	5.2 (3.8–6.6)	3.5 (2.7–4.3)	2.0 (1.4–2.6)	1.3 (0.7–2.0)	1.2 (0.5–2.0)	0.3 (-0.3–0.8)	< 0.1	2.5 (2.1–2.8)
ODD	3.5 (2.3–4.6)	3.0 (2.2–3.7)	2.1 (1.5–2.7)	0.4 (0.1–0.8)	0.2 (-0.1–0.6)	< 0.1	< 0.1	1.9 (1.6–2.2)
TD	3.3 (2.1–4.4)	1.4 (0.9–1.9)	0.5 (0.2–0.8)	0.4 (0.1–0.8)	0.1 (-0.1–0.4)	0.3 (-0.3–0.8)	< 0.1	0.9 (0.7–1.1)
SUD	0.3 (0.0–0.7)	0.4 (0.1–0.6)	0.5 (0.2–0.8)	1.1 (0.5–1.6)	1.7 (0.8–2.6)	3.0 (1.3–4.8)	2.3 (-0.9–5.5)	0.8 (0.6–1.0)

Comorbidities of the several common mental disorders are shown in Table 5. Individuals diagnosed with MDD, AD, OCD and SD had higher rates of comorbidity. MDD was most frequently comorbid with AD (32.8%), OCD (32.5%), and SD (20.0%) in 220, 218, and 134 individuals, respectively. The most common comorbidity of ADHD was AD (29.9%), ODD was ADHD (26.9%), TD was MDD (35.9%) and SUD was SD (28.3%).

**Table 5:** Comorbidity of several common mental disorders

	Disorders population as denominators							
	MDD (%)	AD (%)	OCD (%)	SD (%)	ADHD (%)	ODD (%)	TD (%)	SUD (%)
*Comorbidity number as numerator*								
MDD	–	61.1	32.1	95.7	6.4	14.9	35.9	8.3
AD	32.8	–	19.7	59.3	29.9	23.9	12.7	3.3
OCD	32.5	37.2	–	63.6	3.7	6.0	12.7	23.3
SD	20.0	23.1	13.1	–	5.3	3.0	2.1	28.3
ADHD	1.8	15.6	1.0	7.1	–	26.9	24.6	25.0
ODD	1.5	4.4	0.9	1.4	9.6	–	4.9	6.7
TD	7.6	5.0	2.6	2.1	18.7	10.4	–	3.3
SUD	0.7	0.5	2.1	12.1	8.0	6.0	1.4	–

## Discussion

The mental health of adolescents has become a major public health concern in recent years. Mental disorders are highly prevalent among adolescents and can significantly affect their academic, social, and emotional functioning. Therefore, understanding the prevalence and distribution of mental disorders among them is essential for developing effective prevention and intervention strategies. This study aimed to discuss the findings on the prevalence and distribution of four mental disorders among a large sample of middle school students and examine the gender and age differences in their prevalence rates.

In this study, 2,140 students with mental problems were identified from valid data of 7493 middle school students (detection rate: 28.6%), indicating that more than one in four students were affected by one or more psychiatric disorders. The prevalence rate was generally consistent with data from national and international studies [18, 19], but higher than other studies that screened with the Child Behavior Checklist (CBCL) scale. This difference may be due to the fact that the CBCL is completed by caregivers or students, whereas the MHSC is completed by students only. In addition, the prevalence rate of psychiatric disorders also varied by age group. The prevalence rate was lowest among 12–13 years (23.5%), increased among 14–15 years (29.8%), and was highest among 16–18 years (36.9%), implying that the prevalence of mental disorders increases with age.

What is more, following the MINI-KID interview and DSM criteria for diagnosis, four types of psychiatric disorders were diagnosed in a total of 930 students out of 7943 participants, for an overall point rate of 12.4%. This result is consistent with those reported in epidemiological studies of mental disorders based on a two-stage approach conducted in other countries (7.0–19.8%) [18, 20, 21]. The point prevalence of mental disorders in this study was lower than the pooled prevalence of 13.4% in a meta-analysis. Of these, the prevalence of MDD was higher than the pooled prevalence (8.9% vs. 2.6%), while the prevalence of anxiety was lower (4.8% vs. 6.5%). Gender comparisons showed a higher overall prevalence of psychiatric disorders in girls relative to boys (10.8% vs. 14.0%, *p* < 0.01), contrary to the results of some studies [18, 20, 21]. This result might be related to the inconsistency of the age groups we studied. As other studies have shown, the most common mental disorder among girls is anxiety and depression [19], and in our study, adolescent girls had a higher prevalence of these disorders than boys. However, for disorders, such as ADHD, SUD, TD, SUD and MA, the prevalence was significantly higher in boys than in girls, which was consistent with most studies.

According to the results of two-stage epidemiological survey, there is a general trend of increasing prevalence among students aged 12–18, which may be attributed to the fact that they are in transition from childhood to adulthood, not only going through physical and psychological changes, but also facing other adverse factors that lead to mental health problems, such as academic pressure and family relationships. The 17–18 age group has the highest prevalence rate (21.9% and 21.8%, respectively), which may be related to the transition to university, as they face more difficult courses, and university entrance exams (considered the most important exam of a student’s career in China), with increasing pressure from teachers and parents. On the other hand, we found that the most common comorbidity was MDD rather than ADHD, which is inconsistent with previous findings (children aged 6–16 years) [22]. This may be due to the fact that our survey respondents were between the ages of 12 and 18 years, while the high prevalence of ADHD is between the ages of 6 and 12 years.

Thus, early identification and treatment of psychiatric disorders are essential for preventing negative outcomes, so schools and healthcare providers should screen adolescents for psychiatric disorders and provide appropriate interventions for those who screen positive. Gender and age differences in the prevalence of psychiatric disorders suggest that prevention and intervention strategies should be tailored to specific subgroups of adolescents. For example, targeting girls or older adolescents for prevention and intervention programs may be particularly effective in reducing the prevalence. In addition, the high prevalence of OCD and MDD among adolescents highlights the need for targeted prevention and intervention strategies for these disorders. The findings suggest that prevention and intervention strategies should be tailored to specific subgroups of adolescents and targeted at reducing risk factors and increasing protective factors for specific disorders.

## Conclusion

In conclusion, our study showed that nearly one in ten middle school students in Shaoxing suffer from a mental disorder, with OCD and MDD being the most common disorders in the sample, and the prevalence increases almost with age. The study provides valuable insights into the prevalence of psychological disorders among students and the gender and age differences in their prevalence rates. In addition, our findings highlighted the need for early screening of middle school students for mental disorders, which facilitates the allocation of public mental health resources and policy development. Further research is needed to explore the underlying factors contributing to these gender and age differences and develop effective interventions to improve mental health outcomes in students.

### Limitation

There are several limitations to this study that should be considered when interpreting the findings. First, academic stress and family relationships may be important factors influencing prevalence, but the lack of relevant data collection prevented a correlation analysis in this study. Second, the study did not assess the duration of these mental disorders, which may be important factors in determining the need for intervention. Finally, the study was conducted in a specific geographic region, which may limit the generalizability of the findings to other populations.

## Data Availability

The data sets generated analyzed during the current study are available from the corresponding author on a reasonable request.

## Abbreviations

MHSC: Mental health screening checklist
SCL-90: Symptom inventory 90
SDS: Self-rating depression scale
SAS: Self-rating anxiety scale
Y-BOCS: Yale-Brown obsessive compulsive scale
PSQI: Pittsburgh sleep quality index
MINI-KID: Mini International Neuropsychiatric Interview for Children and Adolescents
DSM-IV: Diagnostic and Statistical Manual of Mental Disorders, Fourth Edition
MDD: Major depressive disorder
AD: Anxiety disorder
OCD: Obsessive–compulsive disorder
SD: Sleep disorder
ADHD: Attention–deficit hyperactivity disorder
ODD: Oppositional defiant disorder
TD: Tic disorder
SUD: Substance use disorder
MA: Mania or hypomania
SOP: Social phobia
PTSD: Post-traumatic stress disorder
BN: Bulimia nervosa
AN: Anorexia nervosa
PD: Psychotic disorders
